# TRIM52 up-regulation in hepatocellular carcinoma cells promotes proliferation, migration and invasion through the ubiquitination of PPM1A

**DOI:** 10.1186/s13046-018-0780-9

**Published:** 2018-06-13

**Authors:** Yi Zhang, Ran Tao, Shan-Shan Wu, Cui-Cui Xu, Jie-Ling Wang, Jie Chen, Yong-Sheng Yu, Zheng-Hao Tang, Xiao-Hua Chen, Guo-Qing Zang

**Affiliations:** 1 Department of Infectious Diseases, Shanghai Jiao Tong University Affiliated Sixth People’s Hospital, 600 Yishan Road, Shanghai, 200233 China; 2 Department of Cardiology, Central Hospital of Minhang District, Shanghai, 201199 China; 3 Department of Critical Care Medicine, The First Affiliated Hospital of Anhui Medical University, Hefei, 230022 Anhui Province China

**Keywords:** Hepatocellular carcinoma, TRIM52, PPM1A, Ubiquitination

## Abstract

**Background:**

Many tripartite motif (TRIM) family proteins have been reported to be of great importance in the initiation and progression in hepatocellular carcinoma (HCC). However, the biological role and regulatory mechanism of tripartite motif containing 52 (TRIM52) in HCC development and progression are poorly defined.

**Methods:**

Immunohistochemistry (IHC), quantitative real-time PCR (qRT-PCR) or Western blot analysis was used to detect TRIM52, p21, matrix metalloproteinase 2 (MMP2), protein phosphatase, Mg^2+^/Mn^2+^ dependent 1A (PPM1A), p-Smad2/3 and Smad2/3 levels in HCC tissues and cell lines. HCC cell proliferation and cell cycle were measured by Cell Counting Kit-8 (CCK-8) and flow cytometry analysis, respectively. HCC cell migration and invasion were measured by Transwell assay. Tumor growth of HCC cells in vivo was measured using the nude mouse xenograft model. The correlation between TRIM52 and PPM1A was measured by co-immunoprecipitation (Co-IP) and ubiquitination analysis in vitro.

**Results:**

TRIM52 was significantly up-regulated in the HCC tissues in comparison with the adjacent non-tumor hepatic tissues. TRIM52 was also up-regulated in HCC cell lines (MHCC-97H and MHCC-97L cells) compared with normal human liver cell line LO2. TRIM52 down-regulation by RNA interfering in MHCC-97H cells enhanced inhibition of cell proliferation, migration and invasion. TRIM52 down-regulation also induced MHCC-97H cells arrest in G0-G1 phase cell cycle and inhibited MHCC-97H cell growth in the nude mice. However, TRIM52 up-regulation in MHCC-97L cells promoted cell proliferation, migration and invasion. Furthermore, TRIM52 down-regulation significantly increased p21 and PPM1A expression, but inhibited MMP2 expression and induced Smad2/3 dephosphorylation in MHCC-97H cells, which were reversed by TRIM52 up-regulation in MHCC-97L cells. TRIM52 was found interacted with PPM1A and TRIM52 down-regulation inhibited the ubiquitination of PPM1A. Importantly, PPM1A up-regulation in MHCC-97L cells significantly suppressed TRIM52-mediated enhancement on cell proliferation, invasion and migration.

**Conclusions:**

Our findings suggest that TRIM52 up-regulation promotes proliferation, migration and invasion of HCC cells through the ubiquitination of PPM1A.

## Background

Hepatocellular carcinoma (HCC) is one of the most common malignant primary liver tumors and is the third leading cause of global cancer death, particularly in East Asia and South Africa [1]. Like other cancers, HCC is involved in a complex and multistep process which is related to a variety of genetic and epigenetic changes. Although many efforts have been made, one of the major problems with HCC is that there are no obvious symptoms, so most patients with HCC are only diagnosed at an advanced stage, and the patients’ survival rates are therefore discouraging [2, 3]. Cirrhosis caused by hepatitis B virus (HBV) or hepatitis C virus (HCV) is reported to be associated with HCC [4]. At present, nonalcoholic fatty liver disease is becoming the leading cause of HCC, and there are no ideal targeted therapies [5]. Additionally, it was also found that oncogenes and/or loss of tumor suppressor genes could be potential biomarkers, as the dysregulated expression level was associated with clinicopathologic characteristics, even prognosis [6, 7]. However, little is known about the molecular and cellular mechanisms. Understanding the mechanisms underlying key genes is essential for further clarifying the pathogenesis of HCC and can provide opportunities for the development of novel therapeutic strategies.

Protein phosphatase, Mg^2+^/Mn^2+^ dependent 1A (PPM1A) is a member of the protein phosphatase 2C (PP2C) family of serine/threonine protein phosphatases, which negatively regulate the cellular stress response pathway [8]. Overexpression of PPM1A can activate the expression of p53 and its downstream p21, which are tumor suppressor genes in cancer development, leading to G2-M phase cell cycle arrest as well as apoptosis, and may be involved in the regulation of tumor pathogenesis [9]. PPM1A can dephosphorylate Smad1, thereby regulating the signaling pathway of bone morphogenetic protein (BMP) in transforming growth factor-β (TGF-β) superfamily [10]. PPM1A can also specifically terminate the TGF-β signaling pathway by dephosphorylation of Smad2/3, which has expanded the substrate type of PPM1A and its signaling pathways [11]. It has been found that PPM1A was downregulated in the HCC tissues compared with the corresponding pericarcinous tissues and that down-regulation of PPM1A through increasing its proteasomal degradation and ubiquitination resulted in the increased ability of HCC cell migration and invasion [12, 13].

Ubiquitination is an important post-translational modification and E3 ubiquitin ligase plays an important role in ubiquitination [14]. Tripartite motif (TRIM) family proteins are considered as E3 ubiquitin ligase with more than 80 members and are associated with developmental disorders, neurodegenerative diseases and viral infections as well as cancer cell growth and differentiation through regulating cell proliferation and apoptosis [15–17]. Tripartite motif containing 52 (TRIM52) is a novel TRIM family protein. A previous study demonstrated that TRIM52 inhibited Japanese encephalitis virus (JEV) replication by degrading the viral nonstructural protein 2A (NS2A) [18]. Additionally, TRIM52 promotes HCC cell proliferation in HBV-associated HCC and HBV X protein (HBx) may regulate TRIM52 expression via the nuclear factor kappa-light-chain-enhancer of activated B cells (NF-κB) signaling pathway [19]. However, the function of TRIM52 in regulating cell cycle and motility of HCC is still largely not understood.

In this study, we have identified that TRIM52 up-regulation in HCC and promote HCC cell proliferation, migration and invasion. Our data suggest that ubiquitination of PPM1A by TRIM52 may be a novel mechanism underlying HCC carcinogenesis.

## Methods

### Study subjects

The human HCC tissue microarrays used in this study were prepared by Shanghai Outdo Biotech Co., Ltd. (Shanghai, China). The clinicopathologic characteristics of the 87 patients enrolled in the study are summarized in Table 1. This study was approved by the Ethics Committee of Shanghai Jiao Tong University Affiliated Sixth People’s Hospital.

**Table 1:** Clinicopathologic characteristics of the patients with HCC

Parameters	Total number of the patients (*N* = 87)
Age (years)	
≤53	47 (54.0%)
> 53	40 (46.0%)
Sex	
Female	8 (9.2%)
Male	79 (90.8%)
Tumor size (cm)	
≤4	25 (28.7%)
> 4	62 (71.3%)
Recurrence (2 years)	
Yes	63 (72.4%)
No	24 (27.6%)
Differentiation	
Low	39 (44.8%)
Moderate	38 (43.7%)
High	10 (11.5%)
Tumor number	
Single	64 (73.6%)
Multiple	23 (26.4%)
TNM stages	
I	15 (17.2%)
II	22 (25.3%)
III	20 (23.0%)
IV	30 (34.5%)
Pathologic stages	
I	6 (6.9%)
II	34 (39.1%)
III	40 (46.0%)
IV	7 (8.0%)
Lung metastasis	
Yes No	16 (18.4%) 71 (81.6%)
HBsAg	
Negative	7 (8.0%)
Positive	80 (92.0%)
HBV infection	
Absent	7 (8.0%)
Present	80 (92.0%)

HBsAg: hepatitis B surface antigen;* HBV*: hepatitis B virus

### Immunohistochemistry (IHC) analysis

After deparaffinization, rehydration and antigen-retrieval, hepatic tissue slides (4–7 μm) were blocked by 3% H_2_O_2_ for 10 min and incubated with anti-Ki67, anti-p-Smad2/3 and anti-MMP2 antibody (Abcam, Cambridge, MA, USA) at 4 °C overnight. The slides were then stained with horseradish peroxidase (HRP)-labeled IgG (Shanghai Long Island Biotec, Shanghai, China) at 25 °C. Subsequently, the sections were stained with diaminobenzidine (DAB), counterstained with hematoxylin and washed in water. The immunoreactive cells were counted in five visual fields of each section under a 200 × light microscope.

### Cell culture and transfection

Human high metastatic HCC cell line MHCC-97H and low metastatic HCC cell line MHCC-97L, and immortalized normal human liver cell line LO2 were obtained from ATCC (Manassas, VA, USA) and cultured in high-glucose Dulbecco’s modified Eagle’s medium (DMEM) (HyClone, Logan, UT, USA) complemented with 10% fetal bovine serum (FBS) (Gibco, Detroit, MI, USA) along with 1% Penicillin-Streptomycin solution separately incubated in 37 °C with 5% CO_2_ and 95% air.

Oligonucleotides encoding short hairpin RNA (shRNA) targeting human TRIM52 (point 670–692, 5’-GGGCATGTGCTTTAAACAC-3′) and scramble shRNA were cloned into the pLKO.1 lentiviral vector. The cDNA encoding TRIM52 and PPM1A was obtained by reverse transcription PCR (RT-PCR) and cloned into pLVX-Puro for constructing pLVX-Puro-TRIM52 and pLVX-Puro-PPM1A expressing vector, respectively. pLKO.1-scramble shRNA (NC) and blank pLVX-Puro (Vector) were used as the negative controls. 293T cells were plated in 6-well plates and transfected with constructs for 4–6 h using Lipofectamine Reagent (Invitrogen, Carlsbad, CA, USA) according to the instructions of the manufacturer. After incubation in a CO_2_ incubator at 37 °C, recombined lentivirus was collected 48 h after transfection and used for MHCC-97H and MHCC-97L cells infection.

### Cell proliferation

After indicated transfection, MHCC-97H and MHCC-97L cells (4 × 10^3^ cells/well) were plated in 96-well plates and cultured for 0, 24, 48 and 72 h, and the cells were added with 10 μL Cell Counting Kit-8 (CCK-8) solution (Signalway Antibody, College Park, MD, USA) with 5% CO_2_ at 37 °C for 1 h, after which the absorbance readings were obtained at 450 nm.

### Cell cycle

Propidium iodide (PI) staining was performed in MHCC-97H and MHCC-97L cells. Briefly, the cells were trypsinized, resuspended in phosphate buffer solution (PBS) containing 10% FBS and fixed in 70% chilled ethanol before cultured in 10 mmol/L RNase at 37 °C for 10 min. After 5 min centrifugation at 1000×g, the cells were resuspended with RNase A (1 mg/mL) for 30 min. Then PI (50 μg/mL) was added in the cells for 10 min at 37 °C under dark. The fluorescence intensities were assessed by flow cytometer (BD Biosciences, San Jose, CA, USA).

### Transwell assay

The invasive ability of MHCC-97H and MHCC-97L cells to pass through filters was measured using a Transwell insert coated with Matrigel. Briefly, MHCC-97H and MHCC-97L cells transfected with indicated lentivirus vectors were serum-starved for 24 h, following which 5 × 10^3^ cells/well in 300 μL serum-free DMEM were placed in the upper chamber at 37 °C. The DMEM medium containing 10% FBS (700 μL) was added into the lower chamber. After 48 h incubation, the cells migrated through to the bottom surface of the membrane were washed with PBS, fixed, stained with 0.5% crystal violet and counted. The invasive cells found on the bottom site of each inserts were then photographed and counted under a microscope (× 200; Olympus, Tokyo, Japan). The migratory ability of MHCC-97H and MHCC-97L cells was measured using Transwell without Matrigel coated.

### Quantitative real-time PCR (qRT-PCR)

Whole RNA was extracted from the HCC cell lines by using TRIzol reagent (Invitrogen) and stored at − 80 °C in RNA secure RNase Inactivation Reagent (Thermo Fisher, St. Louis, MO, USA), and PrimeScript reverse-transcription reagent Kit (Thermo Fisher) was used to carry out reverse transcription reaction on RNA in accordance with the protocols of the manufacturer. Quantitative analysis on the change in expression level was conducted by SYBR Green qPCR Master Mixes (Thermo Fisher) and performed using the GeneAmp PCR System 2700 (Applied Biosystems, Foster City, CA, USA). The primer sequences were shown subsequently:

- TRIM52 forward: 5’-GCCATCTGCTTGGATTACTTC-3′;
- TRIM52 reverse: 5’-TTCATCTTCCTCCTCGTTCTG-3′;
- PPM1A forward: 5’-CCCTTGTTTCCTCTACTTTC-3′;
- PPM1A reverse: 5’-TAATCCTTCCCTACCTATCC-3′;
- GAPDH forward: 5’-AATCCCATCACCATCTTC-3′;
- GAPDH reverse: 5’-AGGCTGTTGTCATACTTC-3′.

The change in the expression of mRNA was assessed by the 2^-ΔΔCt^ approach.

### Western blot assay

Total protein extraction was performed with RIPA Lysis Buffer (Solarbio, Beijing, China) and then centrifuged at 12,000×g for 10 min. Proteins in cell lysates were separated by 10% sodium dodecyl sulfate polyacrylamide gel electrophoresis (SDS-PAGE) and transferred onto nitrocellulose membranes (Millipore, Bedford, MA, USA), then incubated into TRIM52, p21, matrix metalloproteinase 2 (MMP2), PPM1A, p-Smad2/3, Smad2/3 or GAPDH antibodies overnight at 4 °C. The next day, the membranes were washed and subsequently incubated with HRP-conjugated secondary antibodies (1:1000; Beyotime, Nanjing, Jiangsu, China) for 1 h at 37 °C. Then, the membranes were developed using an enhanced chemiluminescence (ECL) detection Kit (Pierce Biotechnology, Rockford, IL, USA).

### Co-immunoprecipitation (Co-IP) and ubiquitination in vitro

Co-IP was performed as described previously [20, 21]. Briefly, cold PBS was used to wash the cells for three times, and the cells were scraped into lysis buffer containing complete protease inhibitors and centrifuged at 14,000×g for 20 min at 4 °C. The supernatants were incubated with normal IgG or anti-HA tag antibody, and the immunocomplexes were then associated with Protein A-Sepharose. Anti-TRIM52 (1:500), anti-PPM1A (1:500), anti-HA-tag (1:500) and normal rabbit/mouse IgG antibody (Santa Cruz Biotechnology, Santa Cruz, CA, USA) were used for Western blot analysis. And the blots were immunoblotted by using anti-HA for ubiquitin.

### Nude mouse xenograft experiment

Twelve nude mice (4–5 week-old BALB/c; Shanghai SLAC laboratory animal Co., Ltd., Shanghai, China) were housed in the animal facility at 25 °C with humidity of 60–70%. MHCC-97H cells (4 × 10^6^) transfected with pLKO.1-shRNA-TRIM52 or pLKO.1-scramble shRNA (NC) were subcutaneously injected into each of the six nude mice, respectively. Tumor volume (mm^3^) was calculated using the following standard formula: (the longest diameter) × 0.5 × (the shortest diameter)^2^. After 33 days, the mice were sacrificed and the tumors were weighted. Care of the laboratory animals and animal experimentation were performed in accordance with the animal ethics guidelines and approved protocols of Shanghai Jiao Tong University Affiliated Sixth People’s Hospital.

### Statistical analysis

Data are presented as mean ± standard deviation (SD), and each test was repeated at least three times. The independent-samples *t* test was applied to two-group analyses, while one-way ANOVA and post hoc Bonferroni test was used when analyzing more than two groups. All statistical analyses were carried out with the GraphPad Prism 6 software (GraphPad Software, San Diego, CA, USA). Two-tailed *P* < 0.05, the difference between groups was considered to be statistically significant.

## Results

### TRIM52 is up-regulated in HCC tissues and cell lines

**Fig. 1:**
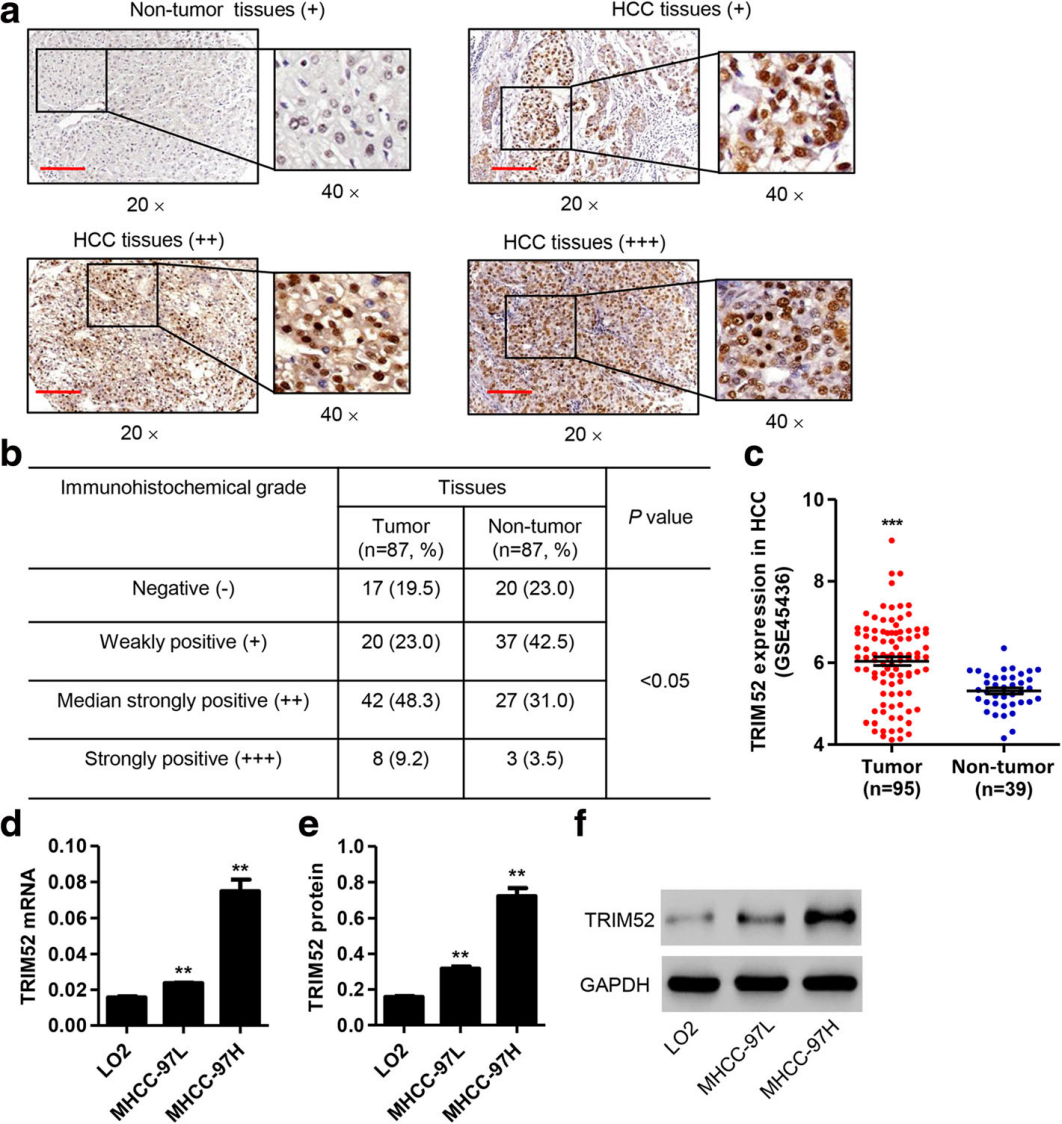
TRIM52 is up-regulated in HCC tissues and cell lines. **a**, **b**. The expression of TRIM52 was measured by IHC analysis on tissue microarrays containing 87 human HCC and adjacent non-tumor hepatic tissues. Scale bars: 100 μm. **c** The expression of TRIM52 based on GSE45436 database containing 95 human HCC and 39 non-tumor hepatic tissues was analyzed. TRIM52 expression in LO2, MHCC-97H and MHCC-97L cells was measured by qRT-PCR (**d**) and Western blot analysis (**e**, **f**), respectively. **P < 0.01 compared with LO2 cells. ***P < 0.001 compared with non-tumor hepatic tissues

IHC analysis on tissue microarrays containing 87 human HCC and adjacent non-tumor hepatic tissue samples found that TRIM52 was significantly up-regulated in the HCC tissues in comparison with the adjacent non-tumor hepatic tissues, specially between groups –, + and groups ++, +++. Median strongly and strongly positive TRIM52 staining was observed respectively in 48.3% (42/87) and 9.2% (8/87) of the HCC tissues (Fig. 1a and b). Moreover, the expression of TRIM52 in HCC based on GSE45436 database was further supported our findings on tissue microarrays (Fig. 1c). Then, we investigated the correlation between TRIM52 expression and clinicopathologic features of the 87 patients with HCC. TRIM52 expression was divided into two levels, low and high, based on the IHC analysis on tissue microarrays. As shown in Table 2, TRIM52 expression was correlated with tumor size, TNM stages and tumor number, but uncorrelated with age, sex, recurrence, pathologic stages, differentiation, lung metastasis, HBsAg and HBV infection.

**Table 2:** The correlation between TRIM52 expression and clinicopathologic features of the patients with HCC

Parameters	TRIM52		*P* value
	Low group, number of the patients	High group, number of the patients	
Age (years)			0.6598
≤ 53	21	26	
> 53	16	24	
Sex			0.7267
Female	3	5	
Male	34	45	
Tumor size (cm)			0.0363
≤ 4	15	10	
> 4	22	40	
Recurrence (2 years)			0.1754
Yes	24	39	
No	13	11	
TNM stages			< 0.0001
I	12	3	
II	14	8	
III	4	16	
IV	7	23	
Pathologic stages			0.2267
I	3	3	
II	18	16	
III	15	25	
IV	1	6	
Differentiation			0.1339
Low	12	27	
Moderate	20	18	
High	5	5	
Tumor number			0.0187
Single	32	32	
Multiple	5	18	
Lung metastasis			0.1164
Yes	4	12	
No	33	38	
HBsAg			0.2247
Negative	5	2	
Positive	32	48	
HBV infection			0.2247
Absent	5	2	
Present	32	48	

Differences between the groups were done by the Chi-square test

HBsAg hepatitis B surface antigen,* HBV* hepatitis B virus

qRT-PCR and Western blot analysis showed that TRIM52 was also up-regulated in HCC cell lines, including MHCC-97H and MHCC-97L cells, compared with normal human liver cell line LO2 (Fig. 1d–f). These data further suggest that TRIM52 is prominently up-regulated in HCC tissues and cell lines and that TRIM52 may facilitate HCC carcinogenesis.

### TRIM52 up-regulation promotes HCC cell proliferation

**Fig. 2:**
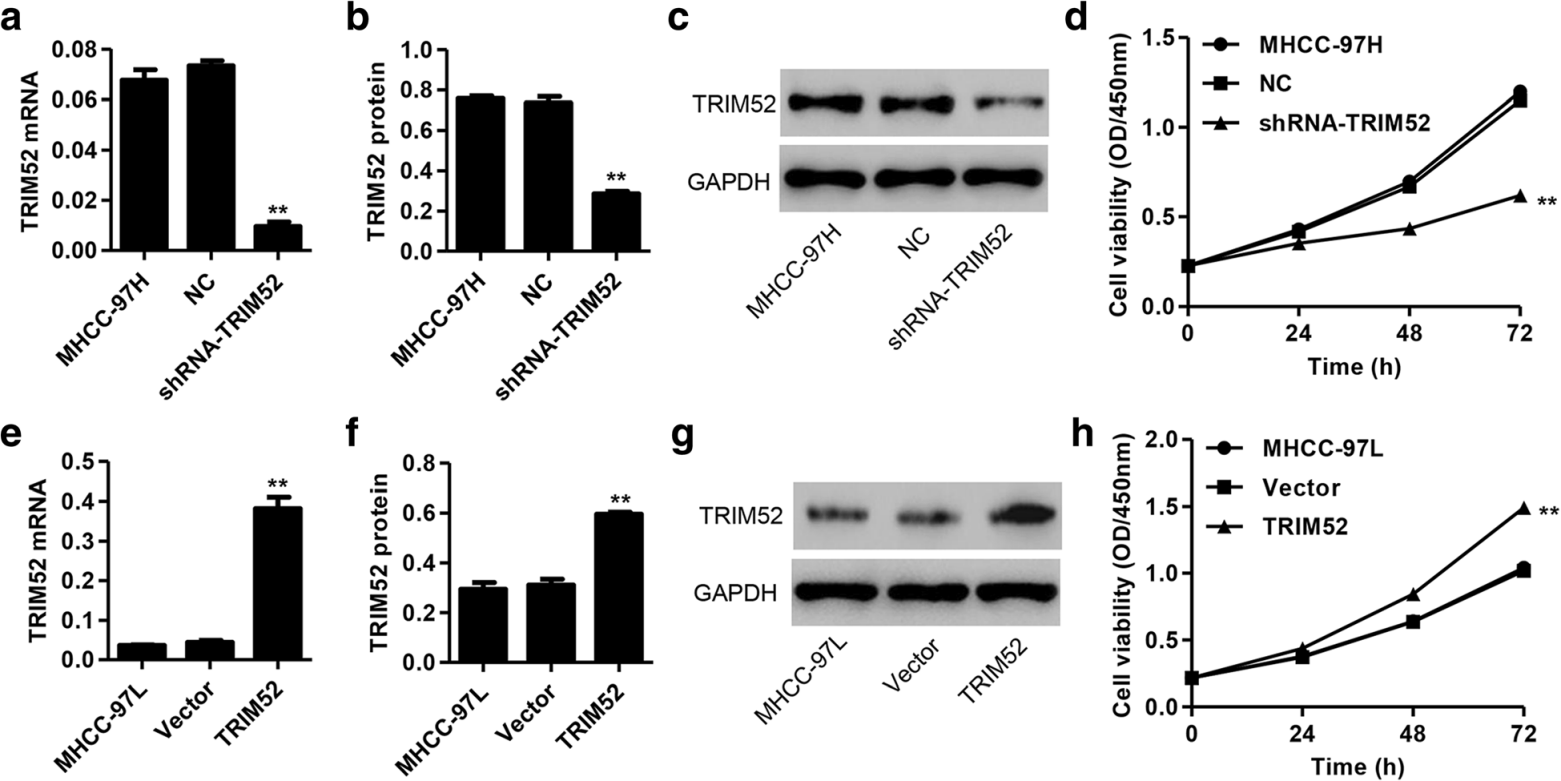
TRIM52 up-regulation promotes HCC cell proliferation. After transfection of MHCC-97H cells with pLKO.1-shRNA-TRIM52 or pLKO.1-scramble shRNA (NC) and MHCC-97L cells with pLVX-Puro-TRIM52 or pLVX-Puro (Vector), TRIM52 expression was measured by qRT-PCR (**a**, **e**) and Western blot analysis (**b**, **c**, **f**, **g**), and the cell proliferation was measured by CCK-8 assay (**d**, **h**). **P < 0.01 compared with corresponding NC or Vector

In order to validate the effects of TRIM52 on HCC cell lines in vitro, shRNA targeting TRIM52 and scramble shRNA were cloned into the pLKO.1 lentiviral vector and transfected into MHCC-97H cells, respectively. Our results showed that there was a significant decrease in the mRNA and protein expression of TRIM52 in MHCC-97H cells with shRNA-TRIM52 transfection compared with scramble shRNA (NC) transfection (Fig. 2a–c). Furthermore, CCK-8 assay demonstrated that shRNA-TRIM52 significantly inhibited the proliferation of MHCC-97H cells by 18.01, 37.67 and 48.33% at 24, 48 and 72 h compared with NC transfection, respectively (Fig. 2d).

Next, TRIM52 encoding sequence was cloned into pLVX-Puro for constructing the pLVX-Puro-TRIM52 expressing vector and transfected into MHCC-97L cells. Blank pLVX-Puro (Vector) was used as the negative control. As shown in Fig. 2e–g, TRIM52 up-regulation significantly increased the mRNA and protein expression of TRIM52 in MHCC-97L cells compared with blank pLVX-Puro (Vector) transfection. Furthermore, CCK-8 assay demonstrated that TRIM52 up-regulation significantly enhanced the proliferation of MHCC-97L cells by 16.03, 31.87 and 43.08% at 24, 48 and 72 h compared with Vector transfection, respectively (Fig. 2h). These results suggest that TRIM52 up-regulation promotes HCC cell proliferation.

### TRIM52 up-regulation promotes cell cycle progress, migration and invasion of HCC cells

**Fig. 3:**
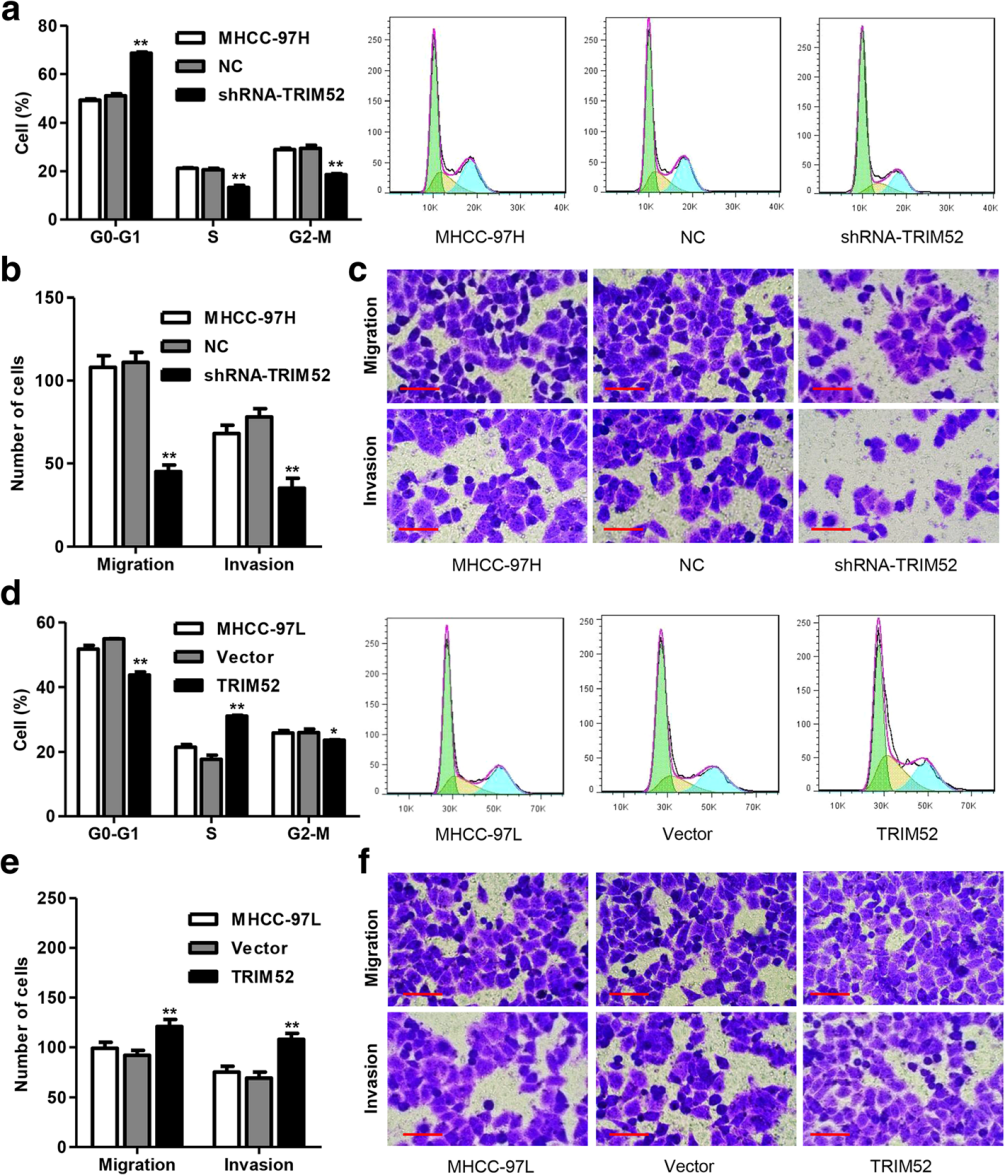
TRIM52 up-regulation promotes cell cycle progress, migration and invasion of HCC cells. After MHCC-97H and MHCC-97L cells transfection as Fig. 2 described, the cell cycle was measured by flow cytometry (**a**, **d**) and the cell migration and invasion was measured by Transwell assay (**b**, **c**, **e**, **f**). Scale bars: 100 μm. *P < 0.05 compared with corresponding NC or Vector. **P < 0.01 compared with corresponding NC or Vector

To further examine the role of TRIM52 in cell growth and motility of HCC cell lines, the cell cycle and migration as well as invasion were measured by flow cytometry and Transwell assay, respectively. We found that TRIM52 down-regulation significantly increased the number of MHCC-97H cells in G0-G1 phase by 34.55% and decreased the number of MHCC-97H cells in S and G2-M phases by 35.32 and 36.89%, compared with NC transfection, respectively, suggesting that TRIM52 down-regulation promoted MHCC-97H cell cycle arrest at G0-G1 phase (Fig. 3a). Moreover, Transwell assay demonstrated that TRIM52 down-regulation significantly inhibited MHCC-97H cell migration and invasion by 59.4 and 55.1% compared with NC transfection, respectively (Fig. 3b and c). Whereas TRIM52 up-regulation significantly increased the number of MHCC-97L cells in S phase by 75.35% and decreased the number of MHCC-97L cells in G0-G1 and G2-M phases by 20.40 and 9.05%, compared with Vector transfection, respectively (Fig. 3d). Furthermore, Transwell assay demonstrated that TRIM52 up-regulation significantly enhanced MHCC-97L cell migration and invasion by 31.5 and 56.5% compared with Vector transfection, respectively (Fig. 3e and f). These results suggest that TRIM52 up-regulation promotes cell cycle progress, migration and invasion of HCC cells.

### TRIM52 up-regulation regulates p21, MMP2, PPM1A, p-Smad2/3 and Smad2/3 expression in HCC cells

**Fig. 4:**
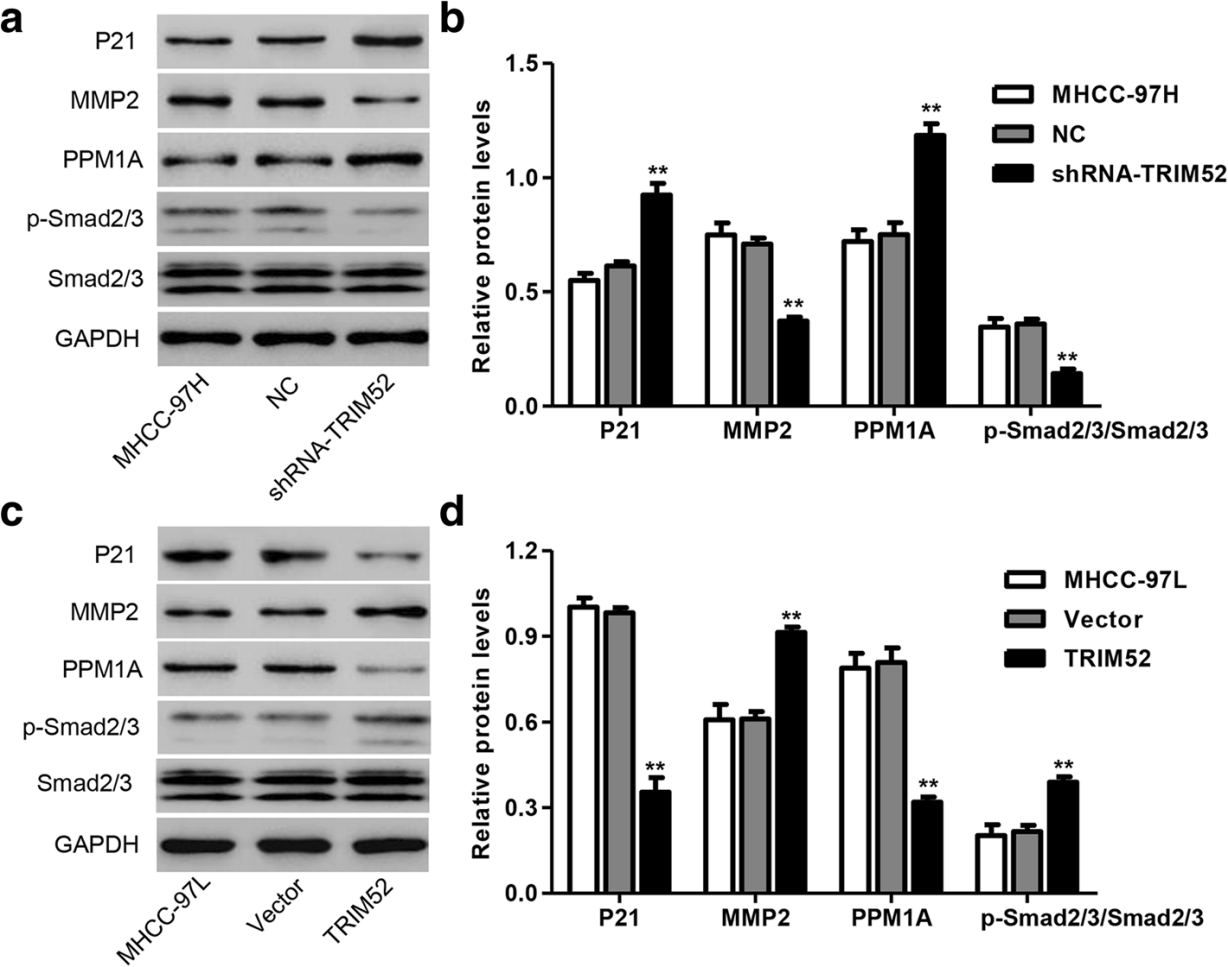
TRIM52 up-regulation regulates p21, MMP2, PPM1A, p-Smad2/3 and Smad2/3 expression in HCC cells. After MHCC-97H (**a**, **b**) and MHCC-97L cells (**c**, **d**) transfection as Fig. 2 described, the expression of p21, MMP2, PPM1A, p-Smad2/3 and Smad2/3 was measured by Western blot analysis. **P < 0.01 compared with corresponding NC or Vector

p21, MMP2, PPM1A, p-Smad2/3 and Smad2/3 expression in HCC cells was also measured by Western blot analysis. As shown in Fig. 4a and b, TRIM52 down-regulation significantly promoted the protein expression of p21 and PPM1A, decreased MMP2 expression and induced Smad2/3 dephosphorylation in MHCC-97H cells compared with NC transfection. However, TRIM52 up-regulation significantly decreased the protein expression of p21 and PPM1A, increased MMP2 expression and induced Smad2/3 phosphorylation in MHCC-97L cells compared with Vector transfection (Fig. 4c and d). These results suggest that TRIM52 up-regulation regulates p21, MMP2, PPM1A, p-Smad2/3 and Smad2/3 expression in HCC cells.

### TRIM52 down-regulation inhibits HCC cell growth, migration and invasion in vivo

**Fig. 5:**
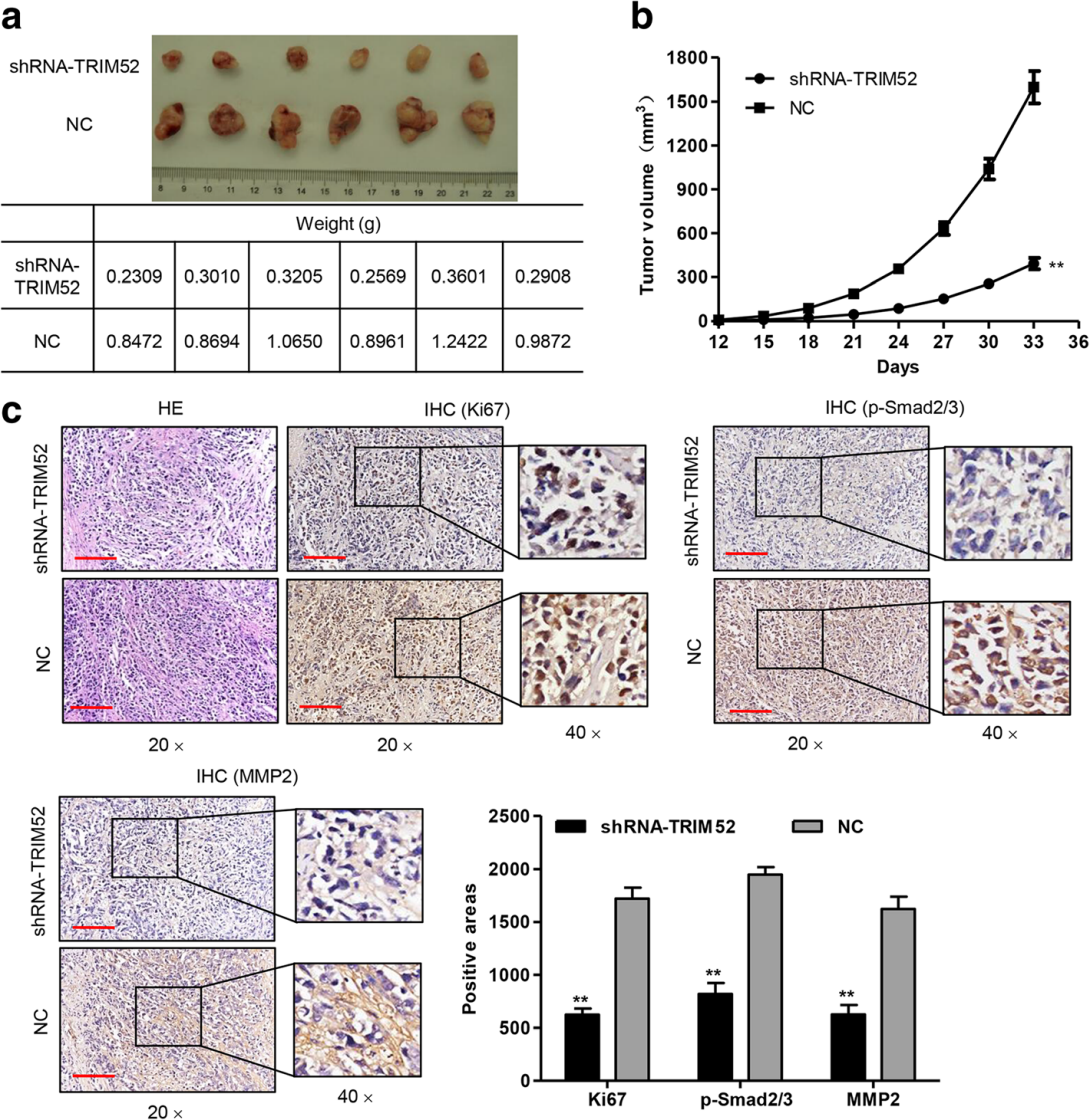
TRIM52 down-regulation inhibits HCC cell growth in vivo. After MHCC-97H cells transfected with pLKO.1-shRNA-TRIM52 or pLKO.1-scramble shRNA (NC) were subcutaneously injected into the nude mice, the tumor weight (**a**), volume (**b**) and Ki67, p-Smad2/3 and MMP2 expression (**c**) were measured, respectively. Scale bars: 100 μm. **P < 0.01 compared with corresponding NC

To confirm the growth effect of TRIM52 in vivo, a xenograft tumor-bearing model was established by inoculating pLKO.1-shRNA-TRIM52 or pLKO.1-scramble shRNA (NC) transfected MHCC-97H cells into the nude mice. Thirty-three days after inoculation the mice were killed and the tumor weights of the pLKO.1-shRNA-TRIM52 transfected mice were significantly decreased compared with those of the NC mice (Fig. 5a). Tumors in the pLKO.1-shRNA-TRIM52 transfected mice grew much slower compared with those in the NC mice (Fig. 5b). Moreover, pLKO.1-shRNA-TRIM52 transfection significantly reduced hepatic injury and Ki67, p-Smad2/3 and MMP2 expression compared with NC transfection measured by hematoxylin-eosin (HE) staining and IHC (Fig. 5c). These results suggest that TRIM52 down-regulation inhibits HCC cell growth, migration and invasion in vivo.

### TRIM52 interacts with PPM1A and TRIM52 down-regulation inhibits the ubiquitination of PPM1A

**Fig. 6:**
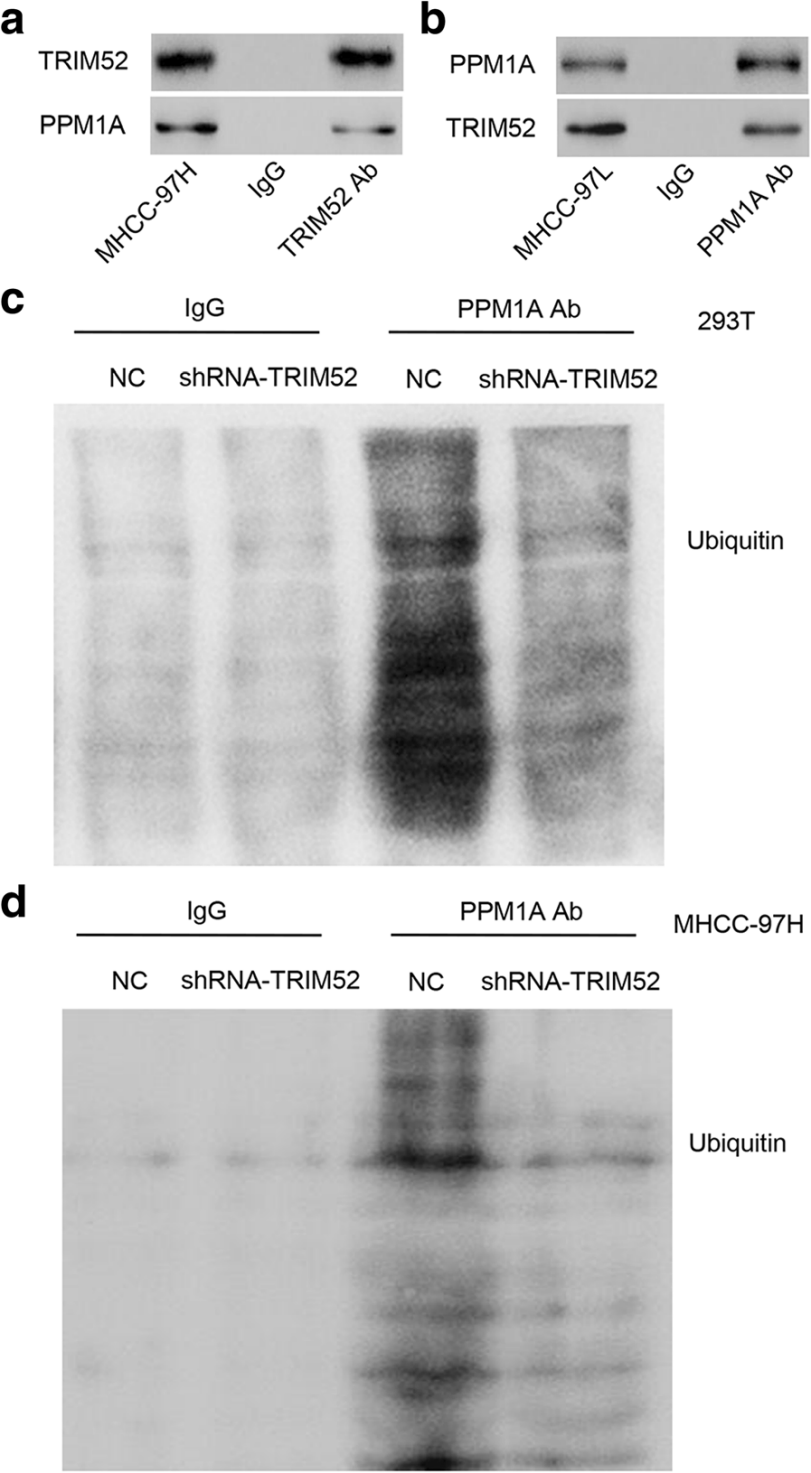
TRIM52 interacts with PPM1A and TRIM52 down-regulation inhibits the ubiquitination of PPM1A. **a**, **b** Co-IP showed that TRIM52 interacted with PPM1A in MHCC-97H and MHCC-97L cells. **c**, **d** PPM1A was immunoprecipitated and immunoblotted in 293T and MHCC-97H cells with pLKO.1-shRNA-TRIM52 or pLKO.1-scramble shRNA (NC) transfection

To elucidate the underlying mechanisms by which TRIM52 exerts its function in HCC carcinogenesis, we identified protein candidates that were functionally associated with TRIM52. PPM1A has been reported to inhibit the Smad2/3 signaling pathway and was significantly modulated by TRIM52 as above described. PPM1A was therefore used for the following analysis. Co-IP assay demonstrated that TRIM52 interacted with PPM1A in MHCC-97H and MHCC-97L cells (Fig. 6a and b). TRIM52 down-regulation significantly inhibited the ubiquitination of PPM1A in 293T and MHCC-97H cells (Fig. 6c and d).

### PPM1A up-regulation inhibits TRIM52-mediated enhancement of cell proliferation, migration and invasion in MHCC-97 L cells

**Fig. 7:**
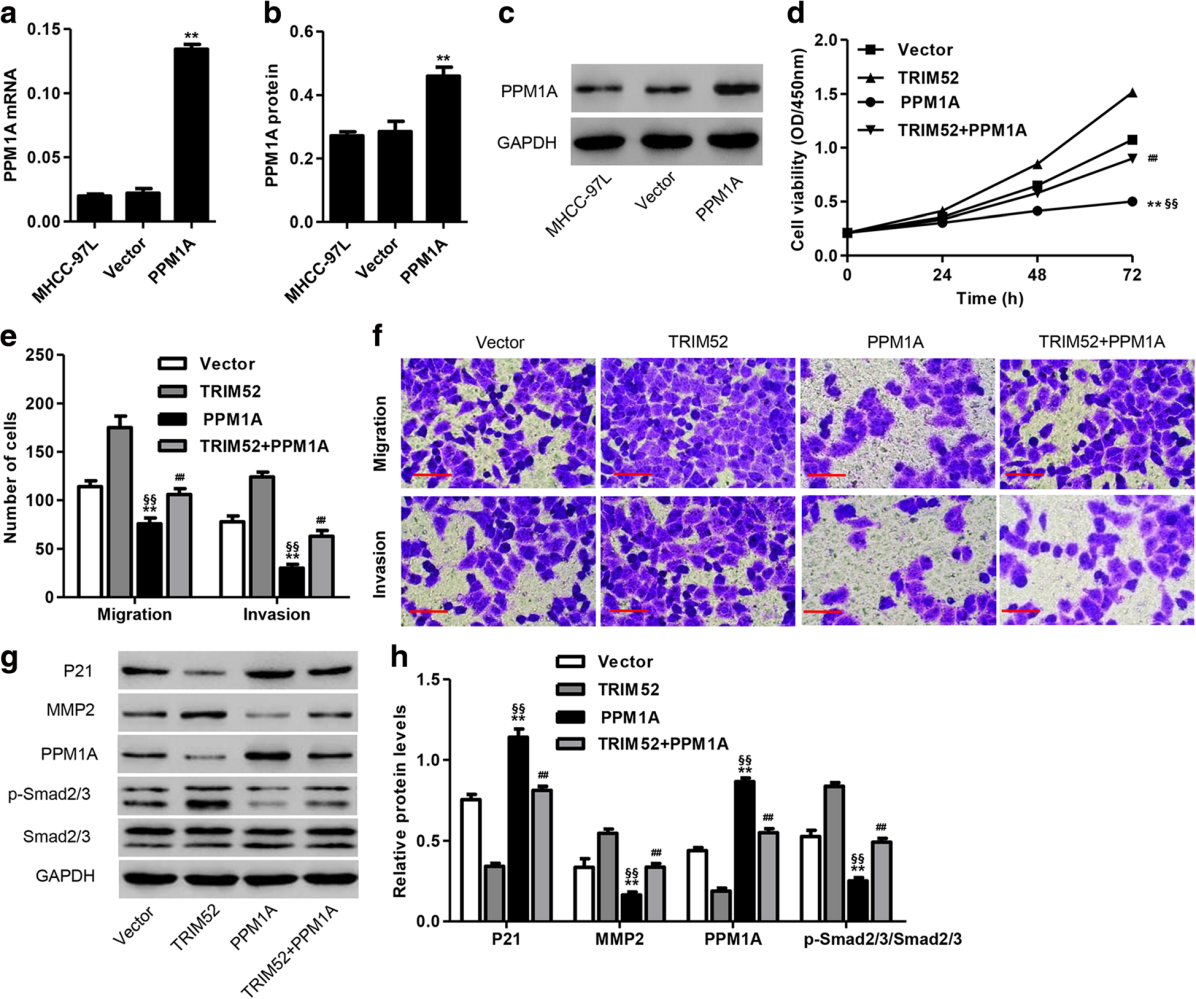
PPM1A up-regulation inhibits TRIM52-mediated enhancement of MHCC-97L cell proliferation, invasion and migration. After transfection of MHCC-97L cells with pLVX-Puro-PPM1A or pLVX-Puro (Vector), PPM1A expression was measured by qRT-PCR (**a**) and Western blot analysis (**b**, **c**). After transfection of MHCC-97L cells with pLVX-Puro-TRIM52 or pLVX-Puro (Vector) in the absence or presence of pLVX-Puro-PPM1A transfection, the cell proliferation was measured by CCK-8 assay (**d**), the cell migration and invasion was measured by Transwell assay (**e**, **f**), and the expression of p21, MMP2, PPM1A, p-Smad2/3 and Smad2/3 was measured by Western blot analysis (**g**, **h**). Scale bars: 100 μm. **P < 0.01 compared with corresponding Vector. ^§§^*P* <0.01 compared with TRIM52. ^##^*P* < 0.01 compared with TRIM52

To further examine the function of PPM1A in HCC carcinogenesis associated with TRIM52, PPM1A encoding sequence was cloned into pLVX-Puro for constructing the pLVX-Puro-PPM1A expressing vector and transfected into MHCC-97L cells. Blank pLVX-Puro (Vector) was used as the negative control. As shown in Fig. 7a–c, PPM1A up-regulation significantly increased the mRNA and protein levels of PPM1A in MHCC-97L cells compared with blank pLVX-Puro (Vector) transfection. Furthermore, CCK-8 assay demonstrated that PPM1A up-regulation significantly inhibited the proliferation of MHCC-97L cells at 24, 48 and 72 h compared with Vector or pLVX-Puro-TRIM52 transfection, respectively. Meanwhile, both TRIM52 and PPM1A up-regulation significantly inhibited the proliferation of MHCC-97L cells at 24, 48 and 72 h compared with only pLVX-Puro-TRIM52 transfection (Fig. 7d). Transwell assay demonstrated that PPM1A up-regulation significantly inhibited MHCC-97L cell migration and invasion compared with Vector or pLVX-Puro-TRIM52 transfection, respectively. Meantime, both TRIM52 and PPM1A up-regulation significantly inhibited MHCC-97L cell migration and invasion compared with only pLVX-Puro-TRIM52 transfection (Fig. 7e and f). Our results also demonstrated that PPM1A up-regulation significantly increased p21 and PPM1A expression, decreased MMP2 expression and induced the dephosphorylation of Smad2/3 in MHCC-97L cells compared with Vector or pLVX-Puro-TRIM52 transfection, respectively. Meanwhile, both TRIM52 and PPM1A up-regulation significantly increased p21 and PPM1A expression, decreased MMP2 expression and induced the dephosphorylation of Smad2/3 in MHCC-97L cells compared with only pLVX-Puro-TRIM52 transfection (Fig. 7g and h). These results suggest that PPM1A overexpression inhibits TRIM52-mediated enhancement of cell proliferation, migration and invasion in HCC cells. PPM1A down-regulation may be an underlying mechanism of TRIM52 in HCC tumorigenesis.

## Discussion

Cellular carcinogenesis is a multistep process involving multiple factors and genes, which is accompanied by changes in a variety of gene expression patterns and which in turn affects the proliferation, apoptosis and differentiation modulated by these genes. The occurrence and development of HCC is also a complex process with multiple genes and steps [22, 23], so it is of great theoretical and practical significance to elucidate the abnormal expression of genes in the process of HCC carcinogenesis. In the present study, we found that TRIM52 was up-regulated in HCC tissues and cell lines. TRIM52 expression was correlated with tumor size, TNM stages and tumor number. Up-regulation of TRIM52 promoted HCC cell proliferation, migration and invasion in vitro and cell growth in vivo through the ubiquitination of PPM1A. Moreover, PPM1A up-regulation inhibited TRIM52-mediated enhancement of HCC cell proliferation, migration and invasion.

A number of recent studies have focused on the function of TRIM proteins in HCC. TRIM3, TRIM16 and TRIM26 down-regulation contributes to poor prognosis in patients with HCC, suggesting the tumor suppressor function in HCC [24–26]. On the contrary, TRIM11 and TRIM31 function as oncogenes of HCC, showing up-regulation in HCC tissues and contributing to HCC cell proliferation and invasion [27, 28]. Except for up-regulation in HCC tissues and cell lines as well as enhancement of HCC cell proliferation reported in our recent study, no other effect has been reported in HCC carcinogenesis involving TRIM52 [19]. In line with the recent findings, our results also demonstrated TRIM52 up-regulation in the HCC tissues compared with the adjacent non-tumor hepatic tissues, and in HCC cell lines, including high metastatic MHCC-97H and low metastatic MHCC-97L, compared with normal human liver cell line LO2. TRIM52 up-regulation promoted HCC cell proliferation, migration and invasion in vitro, and down-regulation of TRIM52 inhibited HCC cell invasion, migration and proliferation, induced G0-G1 phase cell cycle arrest in vitro, and inhibited cell growth and Ki67, p-Smad2/3 and MMP2 expression in vivo.

Next, we studied the mechanism by which TRIM52 regulated HCC carcinogenesis. p21 is considered as a regulator of cell cycle progression at G1 through binding to and inhibiting the activity of cyclin D-CDK2 or cyclin D-CDK4 complexes [29]. Previous studies have indicated that MMP2 activation can enhance invasion, migration and metastasis of HCC [30, 31]. PPM1A down-regulation increased epithelial-to-mesenchymal transition (EMT) progress and invasion through increasing the activity of Smad2/3 signaling pathway in bladder cancer [32]. Simultaneous Smad2/3 phosphorylation was significantly associated with poor outcome of HCC patients after surgery [33] and increased HCC cell growth, invasion and migration [34, 35]. In this study, our results demonstrated that TRIM52 up-regulation inhibited p21 and PPM1A expression, increased MMP2 expression and induced Smad2/3 phosphorylation in HCC cells, which were reversed by TRIM52 down-regulation. These results suggest that these proteins may associate with TRIM52-mediated HCC cell proliferation, invasion and migration.

PPM1A inhibited the invasion and migration of HCC cells and induced the dephosphorylation of Smad2/3 [11–13], suggesting an opposite effect between PPM1A and TRIM52 in HCC. It therefore provides a hypothesis that TRIM52 might involve in HCC carcinogenesis through down-regulating PPM1A. Co-IP and ubiquitination assay confirmed that TRIM52 interacted with PPM1A and TRIM52 down-regulation inhibits the ubiquitination of PPM1A, which was similar to the regulatory mechanism of PPM1A in HBV-related HCC [13]. Moreover, down-regulation of PPM1A via promotion of proteasomal degradation and ubiquitination may result in HCC cell invasion and migration [12]. However, conclusive molecular mechanism by which TRIM52 enhances the ubiquitination of PPM1A need to be further studied. Our experiments demonstrated that PPM1A up-regulation inhibited TRIM52-mediated enhancement of the proliferation, migration and invasion of HCC cells.

## Conclusions

In summary, we present a novel mechanism by which TRIM52 promotes HCC cell proliferation, migration and invasion through ubiquitination of PPM1A. Our study confirms an important function of TRIM52 in HCC development, indicating that strategies to enhance the activity and/or expression of PPM1A can be served as a therapeutic strategy for the prevention and treatment for HCC.

## Abbreviations

BMP: Bone morphogenetic protein
CCK-8: Cell Counting Kit-8
Co-IP: Co-immunoprecipitation
DAB: Diaminobenzidine
DMEM: Dulbecco’s modified Eagle’s medium
ECL: Enhanced chemiluminescence
EMT: Epithelial-to-mesenchymal transition
FBS: Fetal bovine serum
HBV: Hepatitis B virus
HCC: Hepatocellular carcinoma
HCV: Hepatitis C virus
HE: Hematoxylin-eosin
HRP: Horseradish peroxidase
IHC: Immunohistochemistry
JEV: Japanese encephalitis virus
MMP2: Matrix metalloproteinase 2
NC: Negative control
NF-κB: Nuclear factor kappa-light-chain-enhancer of activated B cells
NS2A: Nonstructural protein 2A
PBS: Phosphate buffer solution
PI: Propidium iodide
PP2C: Protein phosphatase 2C
PPM1A: Protein phosphatase, Mg^2+^/Mn^2+^ dependent 1A
qRT-PCR: Quantitative real-time PCR
RT-PCR: Reverse transcription PCR
SD: Standard deviation
SDS-PAGE: Sodium dodecyl sulfate polyacrylamide gel electrophoresis
shRNA: Short hairpin RNA
TGF-β: Transforming growth factor-β
TRIM: Tripartite motif
TRIM52: Tripartite motif containing 52
